# YouTube Videos Related to Skin Cancer: A Missed Opportunity for Cancer Prevention and Control

**DOI:** 10.2196/cancer.4204

**Published:** 2015-03-02

**Authors:** Corey H Basch, Charles E Basch, Grace Clarke Hillyer, Rachel Reeves

**Affiliations:** ^1^ William Paterson University Wayne, NJ United States; ^2^ Teachers College, Columbia University New York, NY United States; ^3^ Mailman School of Public Health Columbia University New York, NY United States

**Keywords:** skin cancer, social media, YouTube

## Abstract

**Background:**

Early detection and treatment influence the mortality risk of skin cancer.

**Objective:**

The objective of this study was to analyze the content of the most viewed professional and consumer videos uploaded to YouTube related to skin cancer.

**Methods:**

A total of 140 professional and consumer videos uploaded between 2007 and 2014 were identified and coded. Coding involved identifying and sorting followed by gathering descriptive information, including length of the video, number of views, and year uploaded. A dichotomous coding scheme (ie, yes or no) was used in coding specific aspects of video content, including provision of information, type of skin cancer, age group, family history, risk reduction, risk factors, fear, and home remedies for skin cancer treatment.

**Results:**

The majority of videos provided information related to screening. Many consumer videos conveyed information related to the use of a black salve as a home remedy for skin cancer, despite the fact that there is no evidence that it is an effective treatment.

**Conclusions:**

Research is needed to identify characteristics of videos that are most likely to be viewed to inform the development of credible communications.

## Introduction

In the United States, skin cancer is the most common cancer affecting both men and women, and incidence rates have recently been rising [1,2]. Early detection and treatment influences mortality risk, particularly with melanoma [3]. Public understanding about the causes, consequences, and treatment of skin cancer may influence individuals’ motivation and ability to make informed decisions regarding prevention, early detection, and treatment. The public has increasingly used the Internet in general and social media in particular as a source of information [4].

YouTube is a popular social media website with approximately one billion unique worldwide users per month [5]. With this extent of reach, there is great potential for both improving understanding or, conversely, creating confusion and disseminating inaccurate and potentially dangerous information. There is limited research on the content of YouTube videos related to public health. In this study, we assessed selected aspects of the most widely viewed YouTube videos related to skin cancer.

## Methods

Using the keywords “skin cancer”, all videos in English were sorted by number of views. Those with 5000 or more views were included in the sample. Each video was classified as being posted from a professional source or consumer. Professional videos were defined as those derived from a health or non-profit organization, or featuring one or more professionals with clinical credentials. Consumer videos featured people with no clinical credentials and the originator was not affiliated with any organization.

A total of 140 professional and consumer videos uploaded between 2007 and 2014 were identified and coded by 1 researcher (RR); 10 were re-coded by 2 researchers (CHB and RR) to demonstrate that the coding was completed in a consistent way. Coding involved an identifying and sorting process followed by gathering descriptive information, including length of the video, number of views, and year uploaded. A dichotomous coding scheme (ie, yes or no) was used in coding specific aspects of video content, including provision of information, type of skin cancer, age group, family history, risk reduction, risk factors, fear, and home remedies for skin cancer treatment.

Descriptive statistics, including frequencies, percentages, means, and standard deviations, were calculated to describe the year each video was uploaded, number of views (since the date of upload), duration (in minutes), and number of views. Chi-square analysis for categorical variables and Student’s *t* test for continuous variables were used to assess if there were differences between videos posted by consumers versus professionals concerning characteristics and content. Interrater reliability was assessed using Cohen’s kappa and was found to be excellent (kappa=.99). *P* values <.05 were considered statistically significant. All analyses were performed using IBM SPSS (version 22).

## Results

Collectively, the 140 videos were viewed more than 33 million times (range 5131-9,049,986 views) (Table 1). Consumers created the majority of videos (60.0%; 84/140). The mean length of the videos was 5 minutes (range: 28 seconds to 86 minutes). There were no statistically significant differences between videos posted by consumers versus professionals with respect to number of videos represented, length, or number of views. There were, however, differences in other respects.

**Figure 1:**
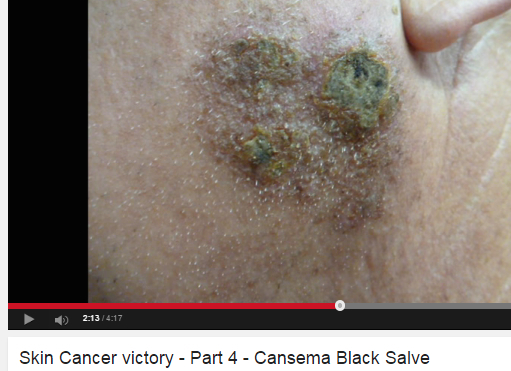
Screenshot for black salve success.

**Figure 2:**
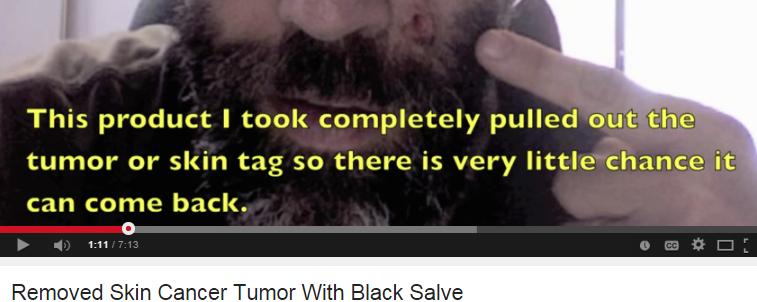
Screenshot of tumor removed.

**Figure 3:**
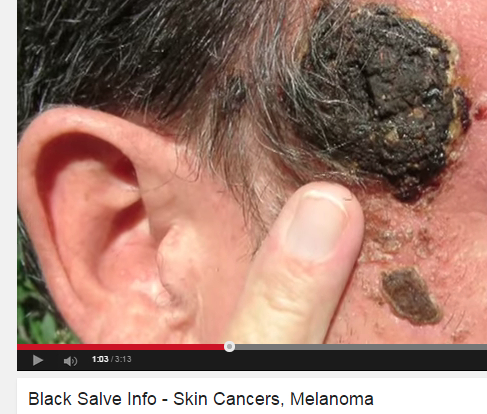
Screenshot of info for the black salve.

The majority of videos (61.4%, 86/140) provided information related to skin cancer screening and tended to discuss skin cancer in general (32.1%, 45/140) or melanoma (26.4%, 37/140) (Table 2). Overall, content was not directed at any specific age group (88.6%, 124/140). Risk reduction was commonly discussed covering signs and symptoms of skin cancer (32.9%, 46/140), importance of screening (28.6%, 40/140), use of sun block (27.9%, 39/140), and dangers of tanning (27.1%, 38/140). Compared with consumer-created videos, those created by professionals more often provided information (*P*<.001), mentioned squamous cell skin cancer (*P*=.02), focused on importance of screening (*P*<.001), and on signs and symptoms (*P*<.001). These videos were also more likely to discuss the ABCDE method of skin cancer self-examination (*P*=.026). Videos created by consumers conveyed information related to the use of black salve as a home remedy cure of skin cancer (consumer 27.7%, 23/83 vs professional 0.0%, 0/57, *P*<.001) (Figure 1-3).

**Table 1:** Characteristics of 140 popular skin cancer screening videos posted on YouTube.

Characteristics		Total (N=140), n (%)	Consumer (N=84, 60.0%), n (%)	Professional (N=56, 40.0%), n (%)	*P* value
**Year video uploaded**					.29
	2007	18 (12.9)	9 (10.7)	9 (16.1)	
	2008	18 (12.5)	8 (9.5)	10 (17.9)	
	2009	15 (10.7)	7 (8.3)	8 (14.3)	
	2010	13 (9.3)	10 (11.9)	3 (5.4)	
	2011	23 (16.4)	18 (21.4)	5 (8.9)	
	2012	26 (18.6)	16 (19.0)	10 (17.9)	
	2013	20 (14.3)	12 (14.3)	8 (14.3)	
	Jan.-Oct. 2014	7 (5.0)	4 (4.8)	3 (5.4)	
**Length of video, minutes**					.16
	Mean (SD)	5.14 (8.75)	4.63 (4.73)	5.92 (12.60)	
	Range	0.28-86.44	0.28-32.23	0.45-86.44	
**Length of video, minutes**					.28
	0.0-1.50	34 (24.3)	22 (26.2)	12 (21.4)	
	1.51-3.20	36 (25.7)	17 (20.2)	19 (33.9)	
	3.21-5.40	35 (25.0)	21 (25.0)	14 (25.0)	
	>5.40	35 (25.0)	24 (28.6)	11 (19.6)	
**Number of video views**					.43
	Total	33,722,068	17,685,501 (52.44)	15,631,764 (46.35)	
	Mean (SD)	237,980 (1,053,305)	210,541 (994,412)	279,138 (1,144,002)	
	Range	5131-9,049,986	5329-9,049,986	5131-7,131,624	

**Table 2:** Content of 140 popular skin cancer screening videos posted on YouTube.

Content		Total (N=140), n (%)	Consumer (N=83), n (%)	Professional (N=57), n (%)	*P* value
Provide information		86 (61.4)	33 (39.8)	53 (93.0)	<.001
**Type of skin cancer**					
	Brief mention or irrelevant	11 (7.9)	10 (12.0)	1 (1.8)	.026
	Melanoma	37 (26.4)	19 (22.9)	18 (31.6)	.25
	Basal cell carcinoma	25 (17.9)	17 (20.5)	8 (14.0)	.33
	Skin cancer in general	45 (32.1)	30 (36.1)	15 (26.3)	.22
	Squamous cell carcinoma	13 (9.3)	4 (4.8)	9 (15.8)	.03
	Multiple types	16 (11.4)	7 (8.4)	9 (15.8)	.18
**Age group discussed**					.016
	Age not discussed	124 (88.6)	79 (95.2)	45 (78.9)	
	<40	4 (2.9)	1 (1.2)	3 (5.3)	
	≥40	4 (2.9)	0 (0.0)	4 (7.0)	
	All ages	8 (5.7)	3 (3.6)	5 (8.8)	
Family history		16 (11.4)	6 (7.2)	10 (17.5)	.06
**Risk reduction**					
	Importance of screening	40 (28.6)	12 (14.5)	28 (49.1)	<.001
	Signs and symptoms	46 (32.9)	16 (19.3)	30 (52.6)	<.001
	Use of sunblock	39 (27.9)	24 (28.9)	15 (26.3)	.74
	Danger of tanning	38 (27.1)	20 (24.1)	18 (31.6)	.33
	Wearing a hat	13 (9.3)	8 (9.6)	5 (8.8)	.86
	Prevention in youth	12 (8.6)	6 (7.2)	6 (10.5)	.49
	ABCDE method	15 (10.7)	5 (6.0)	10 (17.5)	.03
Fear		19 (13.6)	14 (16.9)	5 (8.8)	.17
**Home remedies for skin cancer treatment**					
	Black salve	23 (16.4)	23 (27.7)	0 (0.0)	<.001

## Discussion

### Principal Findings

This is the first study to examine the content of videos pertaining to skin cancer on the popular social media site, YouTube. It is important for health professionals to recognize that a great deal of information accessed by the public is from material posted by consumers. Indeed, consumers posted the majority of the most widely viewed videos related to skin cancer prevention and treatment. Perhaps the most important finding from this study is the focus of consumer videos on home remedies for skin cancer, namely the use of black salve for treatment or removal of cancers on the skin.

Black salve varies in composition but typically contains zinc chloride and/or powdered bloodroot from *Sanguinaria canadensis* [6]*.* There is no evidence that black salve is effective in treating skin cancer. Two case studies were identified in which patients attempted to use black salve for treatment of skin cancer, but patients’ melanoma in these studies persisted [7,8]. More than one in four consumer videos (27.7%, 23/83) focused on black salve, and these videos were viewed over 3 million times. Videos can be particularly deceiving as they tend to show before and after imagery and messages tend to be delivered from a person claiming to have used the product with success.

Additionally, none of these popular videos were posted by a US governmental health agency. Given that prevention and control of skin cancer is a goal of multiple agencies of the US Public Health Service as well as non-profit agencies, the lack of widely viewed communications on this topic represents a missed opportunity for disease prevention and health promotion. The number of views was sizeable, though it is not distinguishable whether the views represent unique users.

YouTube has proven to be a valuable tool for health information in the digital age. The medium has been used for a wide range of purposes, including the creation of educational materials for health care professionals [9], the generation of a patient community where discussion about experiences and treatment can occur [10], the documentation of patient experiences for side-by-side health communication messages [11], and personal barriers to accessing care [12]. The platform provides a unique mix of social media and visual representation that can create confusion in regard to the quality of health information available. One study reported on the unreliability and misleading nature of the information presented on the website [13], while another has shown that the majority of condition specific videos are generally consistent with medical recommendations [11].

### Conclusion

Additional research is needed to identify the characteristics of videos that are most likely to be viewed and to develop credible communications through YouTube and other social media to help the public make informed decisions about cancer prevention and control.
